# Brown bear (*Ursus arctos*) attacks resulting in human casualties in Scandinavia 1977–2016; management implications and recommendations

**DOI:** 10.1371/journal.pone.0196876

**Published:** 2018-05-23

**Authors:** Ole-Gunnar Støen, Andrés Ordiz, Veronica Sahlén, Jon M. Arnemo, Solve Sæbø, Glenn Mattsing, Magnus Kristofferson, Sven Brunberg, Jonas Kindberg, Jon E. Swenson

**Affiliations:** 1 Norwegian Institute for Nature Research, Trondheim, Norway; 2 Faculty of Environmental Sciences and Natural Resource Management, Norwegian University of Life Sciences, Ås, Norway; 3 The Norwegian Environment Agency, Trondheim, Norway; 4 Department of Wildlife, Fish and Environmental Studies, Swedish University of Agricultural Sciences, Umeå, Sweden; 5 Department of Forestry and Wildlife Management, Faculty of Applied Ecology and Agricultural Sciences, Inland Norway University of Applied Sciences, Koppang, Norway; 6 Faculty of Chemistry, Biotechnology and Food Science, Norwegian University of Life Sciences, Ås, Norway; 7 Rovdjurscentret De 5 Stora, Järvsö, Sweden; 8 Swedish Environmental Protection Agency, Stockholm, Sweden; 9 Swedish Association for Hunting and Wildlife Management, Öster Malma, Sweden; Université de Sherbrooke, CANADA

## Abstract

Human persecution and habitat loss have endangered large carnivore populations worldwide, but some are recovering, exacerbating old conflicts. Carnivores can injure and kill people; the most dramatic form of wildlife-human conflict. In Scandinavia, the brown bear (*Ursus arctos*) population increased from ~500 bears in 1977 to ~3300 in 2008, with an increase in injuries, fatalities, and public fear of bear attacks. We reviewed media coverage and interviewed victims to explore how bear population trends, hunter education, and other factors may have influenced the number of injuries and fatalities in Scandinavia from 1977 to 2016. We found 42 incidents with 42 injuries and 2 fatalities; 42 were adult men, one was an adult woman conducting forestry work, and one was a boy skiing off-piste. Thirty-three adult men were hunting bears, moose, or small game, often with a hunting dog, and 26 had shot at the bear at 8±11 m before injury. Eleven nonhunters were conducting forestry work, inspecting a hunting area, picking berries, tending livestock, hiking, harassing a denned bear, and one person was killed outside his house at night. Eight of the 11 incidents of nonhunters involved female bears with cubs; three of these family groups were in dens and two were on carcasses. The annual number of hunters injured/killed was mostly influenced by the increase in the bear population size. The pattern was similar regarding injuries/fatalities to other outdoor users, but the relation with the bear population size was weaker than for hunters, and the null model was equally supported. Bear physiology at denning may make encounters with bears more risky in the fall, when bears show prehibernation behavior. Awareness and education efforts, especially among hunters, seem important to ensure human safety. Recreationists and forestry workers should avoid dense vegetation or make noise to warn bears of their presence.

## Introduction

Large carnivores facilitate biodiversity [1–3], but they can threaten human safety by injuring or killing people. This is the most dramatic form of wildlife-human conflict, a source of public opposition to large carnivore conservation, and a challenge for carnivore management [4–7]. Human persecution and habitat loss have endangered large carnivore populations worldwide [8,9], but some are recovering, exacerbating old conflicts [10,11]. Historically, attacks on humans have been a major source of human conflict with large carnivores [12] and increasing rates of attacks by some species [7,13] undermine large-carnivore protection efforts and population recovery on several continents. The issue is therefore of broad concern for conservation and management, in addition to human safety.

Although more people are killed by species such as domestic dogs, horses, cattle, or snakes than by bears [14,15], the attitudes of people towards bears and other large carnivores reflect their concerns about personal safety. Rareness of attacks also contribute to generate much media attention, which can influence people’s attitudes [16,17]. How people perceive the risk of attack affects the tolerance they have for conservation efforts [18–20] and, although efforts have been made to try to reduce human fear of large carnivores [21], it is essential to provide managers and the public with accurate information regarding the risks that carnivores pose to people in order to reduce these risks.

Here we present the case of the Scandinavian brown bear (*Ursus arctos*) population, which increased to ~3300 bears by 2008 in Sweden [22], where > 95% of Scandinavian bears live, after near extirpation around 1930 [23]. Nowadays there are ~2800 bears in Sweden [24]. Injuries from bear attacks have increased during the last decades and fatalities have been documented for the first time for more than 100 years [15,25]. Repeat questionnaires reveal that public fear of large carnivores is increasing [26,27].

Brown bears are hunted in Scandinavia [28] and hunters have been the most affected by bear attacks, compared to other outdoor users [15]. After a fatal incident in 2004, hunters in Sweden focused on dangers associated with hunting bears and hunting in areas with bears, resulting in information campaigns and, since 2007, annual hunting courses focusing on bear behavior and safety when encountering bears. In this paper we review all incidents where bears have caused human injuries or fatalities in Scandinavia from 1977 to 2016 and evaluate how brown bear population trends, number of hunters and nonhunter outdoor users, education of hunters, and other risk factors based on bear ecology may have influenced the number of injuries and fatalities in the last four decades. Our goal is to provide information that can help avoid new incidents, which is both of management and conservation interest.

## Methods

We reviewed newspapers, hunting magazines, books, and scientific publications for human injuries or fatalities caused by brown bears in Scandinavia during 1977–2016. We interviewed the injured people to confirm or supplement the written information (one had died before our study) and reviewed the police reports about the two fatalities, which are also described in detail elsewhere [25]. The research procedure for humans was presented to the Ethical Review Board in Sweden, which declared that the study needed no further ethical review. We define an injured person as one who received medical treatment, and use the term “incident” as a situation where a bear caused one or more injuries or fatalities. We first summarized the factors involved in the incidents, recording the following variables: year, date, time of the day, location, number, gender, and age of the affected people, their primary activity, whether or not they shot at the bears before being injured or killed and the shooting distance, distance to the bears when first observed, number of bears, sex and age of the bears, and whether dogs were involved and their behavior towards the bears.

We used generalized linear models (Poisson regression) in R [29] to test whether the annual estimates of the bear population size, the annual bear harvest in Sweden, the number of Swedish hunters and outdoor users, and the cumulative number of hunters attending annual hunting courses arranged by the Swedish Association for Hunting and Wildlife Management since 2007 were related to the number of combined injuries and fatalities annually during 1977–2016. We ran the models separately for hunters and nonhunters, as the circumstances around the attacks could be different between these groups. We tested candidate models for each response variable and ranked the models in terms of the corrected Akaike Information Criterion (AICc) [30,31], interpreting the importance of parameters with the 95% confidence intervals (CI) of their effect estimates e.g. [32].; 95% CI that included 0 were not informative, because the direction of the effect was unclear.

**Fig 1:**
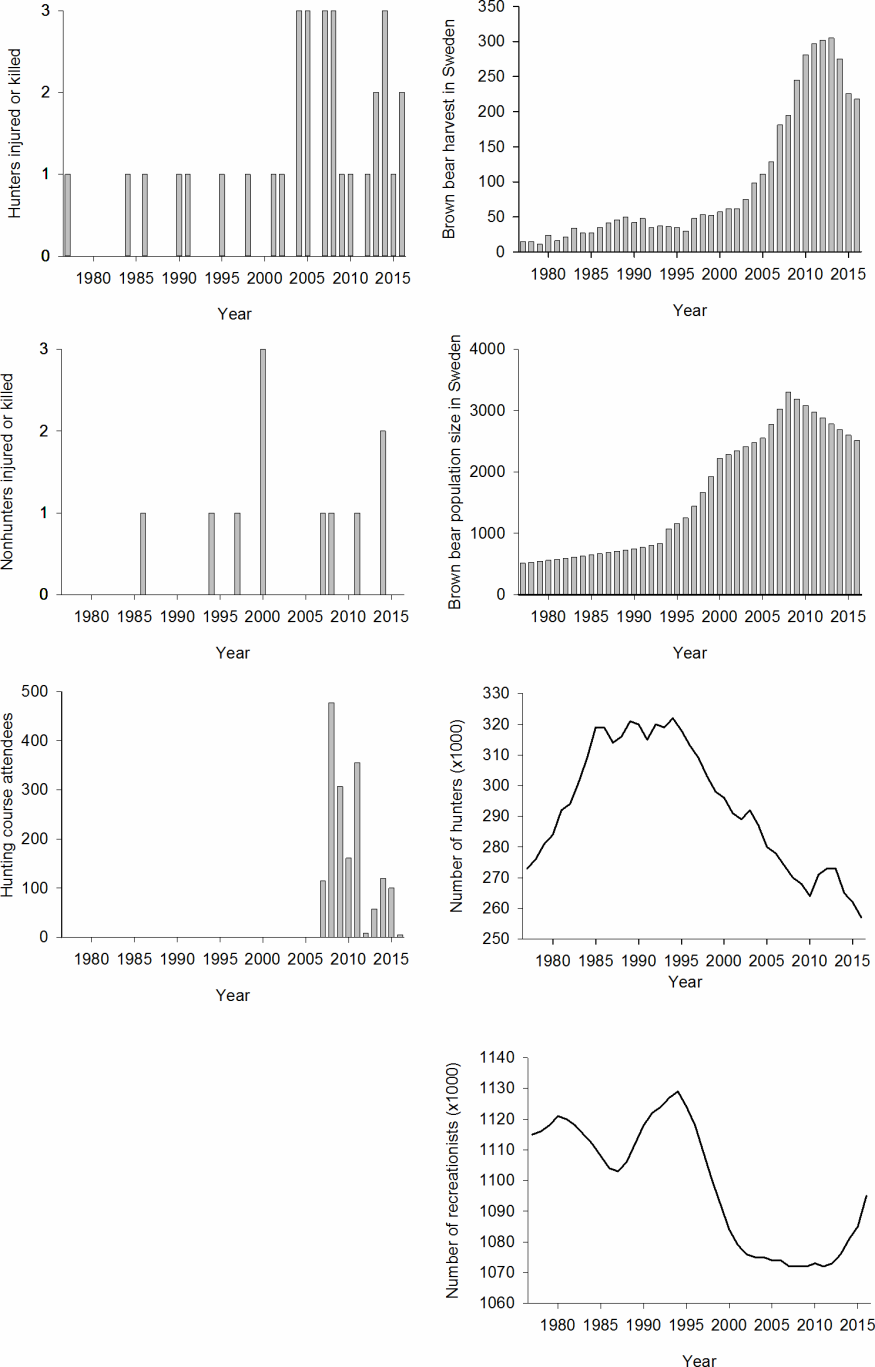
Annual numbers of brown bears, outdoor recreationists, and bear-caused human injuries/fatalities in Scandinavia during 1977–2016. (A) Number of hunters injured/killed by a bear. (B) Number of nonhunters injured/killed by a bear. (C) Number of hunters attending hunting courses that teach about bear behavior and safety when encountering bears. (D) Number of bears harvested during the brown bear hunting season in Sweden. (E) The estimated brown bear population size in Sweden. (F) Number of hunters in Sweden. (G) Number of outdoor recreationists in Swedish counties with bears.

The bear population size and number of bears harvested were highly correlated (Pearson’s correlation coefficient = 0.83), because the annual harvest quota followed the trend of the bear population (Fig 1D and 1E). We used bear population size in the final set of candidate models, because it was more explanatory than the number of harvested bears. The annual bear population estimates were created from scientifically published estimates that are summarized in [24], and calculations of normal growth curves for the intervening years, using the equation: r = ln(nt+x/nt)/x, where r is the instantaneous rate of increase, n is the population size estimate in a year t, and x is the number of years between estimates. Estimates for 1991, 1993, and 1996 were corrected by adding 24.6%, due to a documented underestimation of the method of estimation used during those years [33]. The annual number of Swedish hunters and the number of those attending hunting courses were provided by the Swedish Association for Hunting and Wildlife Management and the Swedish Environmental Protection Agency. In Sweden, there are no specific bear licenses and anyone with a hunting license, appropriate weapon, and right to hunt in the area can shoot one or more bears [28], if the annual quota in the area has not been filled. The annual bear harvest was provided by the National Veterinary Institute in Sweden and the Swedish Environmental Protection Agency. The number of nonhunting outdoor users was estimated from the annual human population that lives in counties within the bear range in Sweden (Statistics Sweden, http://www.scb.se/en/). During our study period, the proportion of people participating at least once per year in outdoor activities, such as hiking, has been stable (70–80% of the total human population; [34]), which allowed us to estimate a minimum number of outdoor users on an annual basis, assuming that 75% of the human population was outdoors at least once annually.

## Results

**Fig 2:**
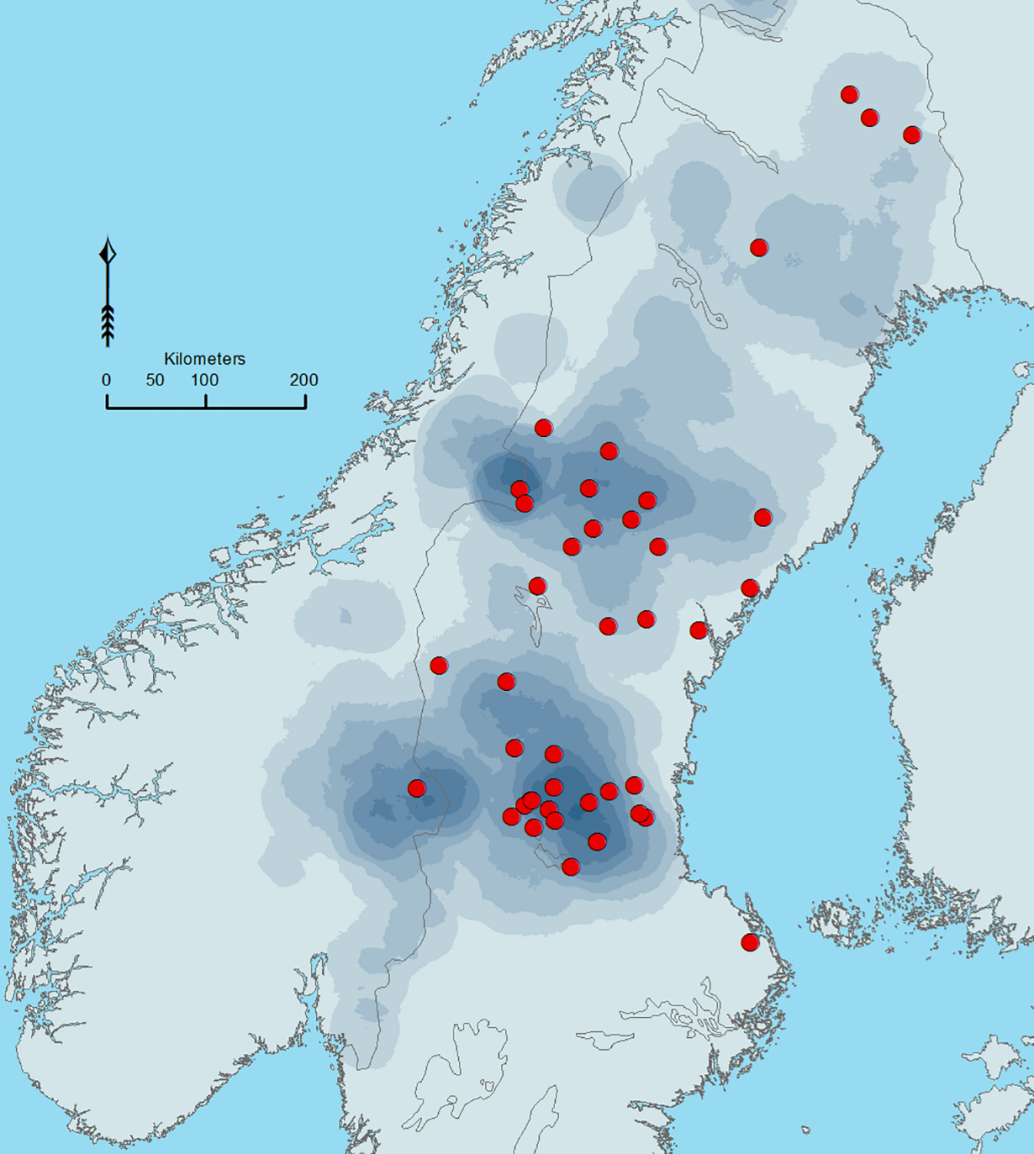
Location of brown bear-caused human injuries/fatalities in Scandinavia 1977–2016. Red dots show where persons were injured or killed by a brown bear and the shades indicate relative bear density (darker shade means higher density), based on densities of bear scats collected in Sweden and Norway for monitoring purposes during 2001–2012.

Forty-four people were injured (n = 42) or killed (n = 2) by brown bears in 42 incidents in Scandinavia during 1977–2016 (Figs 1 and 2). During this period, the bear population in Sweden increased six fold, from ~500 in 1977 to ~3300 individuals in 2008, and harvest also increased six fold, in response to a corresponding increase in the quota levels, from ≤50 before 1998 to a maximum of ~300 in 2013 (Fig 1D and 1E), despite the decrease in the total number of hunters during the second half of our 40-year study period (Fig 1F). All injuries/fatalities of hunters were adult men (n = 33), which were hunting moose *Alces alces* (n = 18), bears (n = 13), or small game (n = 2). Of the 11 injured nonhunters, nine were adult men. Of these, two were conducting forestry work, two were inspecting a hunting area, one was picking berries, one was hiking, one was tending livestock, one was harassing a denned bear, and one person was killed outside his house at night. One woman was injured during forestry work and a 12-year-old boy was injured when he fell into the den of a female bear with three cubs while downhill skiing off-piste.

Most (84%) of the incidents involving hunters occurred between 0800 and 1600 (Table 1). Eighteen of the 33 injured hunters (55%) were hunting moose. Dogs harassed the bear in 77% of all the incidents involving hunters. Hunters saw the bear for the first time at an average distance of 15±19 m (SD; n = 30). In 73% (n = 22) of the incidents, the hunters shot at the bear before being injured, which also occurred in 2 of the 11 incidents involving armed, nonhunters. Although armed, they were not hunting; one was inspecting a hunting area and one was attending livestock. Injured bear hunters shot at the bear before being injured in 83% of the incidents, whereas injured hunters pursuing other game shot at the bear before being injured in 67% of the cases. The mean distance between the hunter and the bear when the first shot was fired was 8±11 m (n = 24).

**Table 1:** Summary of information on bear attacks by brown bears that resulted in injuries or death of hunters and nonhunters, in Scandinavia, 1977–2016.

	Time 08:00–16:00	Hunting moose	Dog harassed bear	Distance to seen bear (±SD)	Shot at bear before injury	Distance to bear at first shot (±SD)	Female bears with cubs	Bears at dens
Hunters	84%	55%	77%	15±19 m	73%*	8±11 m	19%	39%
Nonhunters	90%	-	18%	11±9 m	-	-	73%	27%

* 83% bear hunters; 67% other hunters

**Fig 3:**
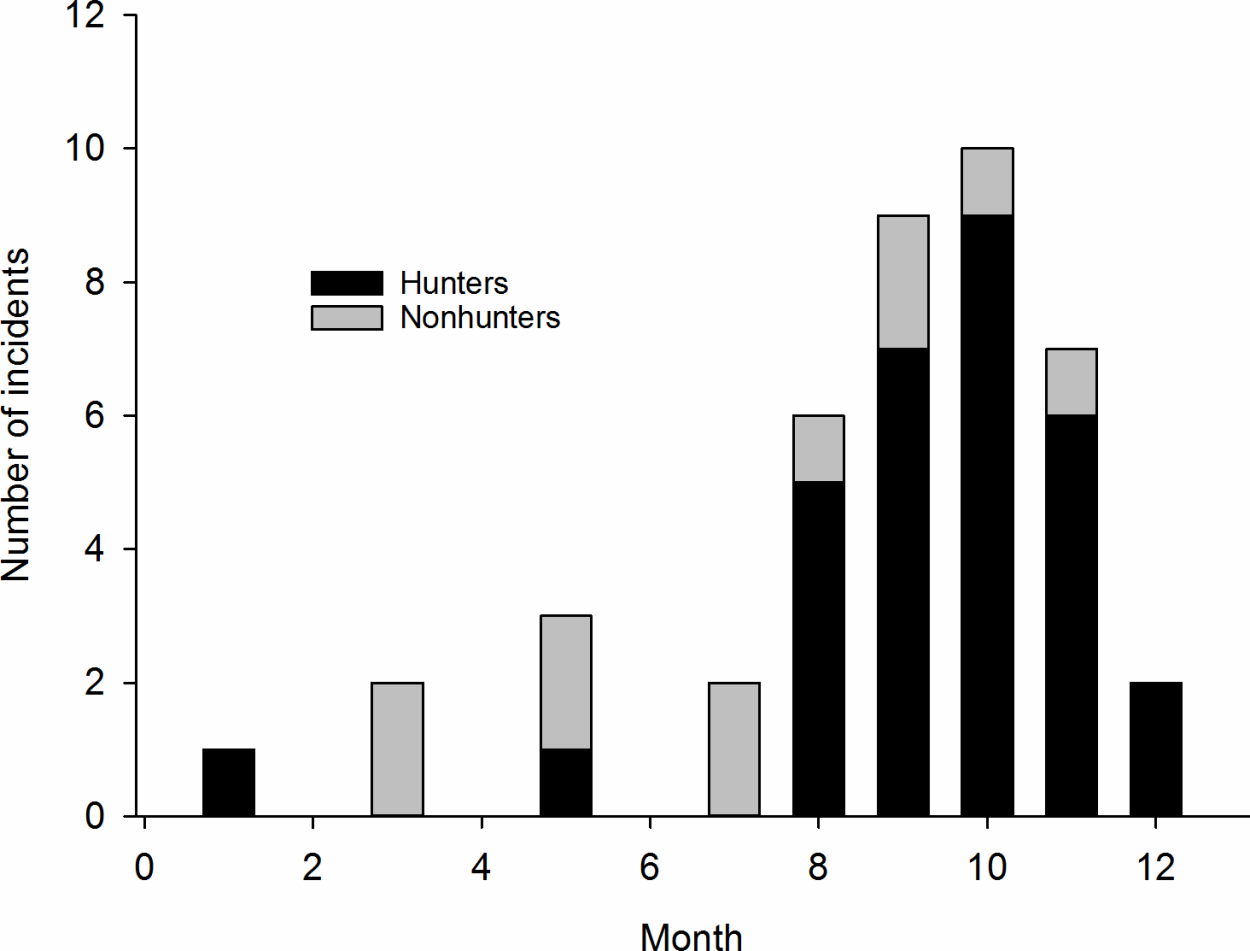
Monthly distribution of brown bear-caused injuries/fatalities of hunters and nonhunters in Scandinavia during 1977–2016.

Eighteen incidents involving hunters (58%) occurred during the months of October through January, whereas 9 incidents (82%) involving nonhunters occurred during March through September (Fig 3). Nine of 10 nonhunter injuries with known time of day occurred between 0800 and 1600 and injured nonhunters first observed the bears at shorter distances, 11±9 m, than the 15±19 m for hunters. Eight of the 11 incidents (73%) involving nonhunters were caused by female bears with cubs; three of these family groups were in dens and two were on a carcass. Two cases were male bears on a carcass and a single female in a den. Six of the 31 incidents (19%) with hunters involved females with cubs. Bears at dens were involved in 3 of 11 incidents with nonhunters (27%) and 12 of 31 with hunters (39%). The distance when the bear was observed by those three injured nonhunters was 1.5±0.9 m, while the bears were first observed at 9±6 m by the injured hunters with dens involved.

The increase in combined bear-caused injuries and fatalities in 1977–2016 was best explained by the increase in the number of injured/killed hunters (33 of the 44 injured/killed). The annual number of hunters injured/killed was mostly influenced by the increase in the bear population size (Table 2). The second-best model (ΔAICc = 1.56) included the bear population size and a negative, but non-significant relation (95% CI of the parameter estimate included 0) between the number of hunters injured/killed and the trend in the number of hunters (Table 2).

**Table 2:** Statistical results for brown bear-caused human injuries/fatalities in Scandinavia in 1977–2016. Results from generalized linear models (GLM) using Poisson link function explaining the annual number of hunters injured/killed by bears and the annual number of nonhunters injured/killed.

	AICc	Delta	Model weight (%)	Effect estimate	95% CI
**Models for hunters**					
1. Incidents hunters ~ bear population size	87.3	0	44		
parameter: bear population size				0.0008	(0.0004, 0.001)
2. Incidents hunters ~ bear population size, number of hunters	88.9, 1	1.56	20		
parameter: bear population size				0.0006	(0.00005, 0.001)
parameter: number of hunters				-0.00001	(-0.00004, 0.00002)
3. Null model	102.9	15.5	0		
**Models for nonhunters**					
1. Incidents nonhunters ~ bear population size	55.8	0	30		
parameter: bear population size				0.0006	(0.000005, 0.001)
2. Incidents nonhunters ~ number of outdoor users	56.9	1.09	18		
parameter: outdoor users				-0.00003	(-0.000006, 0.000004)
3. Null model	57.5	1.7	13		

The annual number of bear-caused injuries/fatalities of nonhunters was also related to the increasing number of bears, but the null model (ΔAICc = 1.7) was equally supported (Table 2). A second supported model (ΔAICc = 1.1) suggested a negative relation between the number of injuries/fatalities of nonhunters and the total number of outdoor users, but the 95% CI for this parameter included 0 (Table 2).

In summary, the only well supported model showed a positive relation between the number of bear-caused injuries/fatalities to hunters and the increasing bear-population size, whereas other parameters had smaller effects with unclear directions (95% CI of the effect estimates included 0). We used Poisson models in our analyses, therefore the effect of the bear population size on the expected number of injuries/fatalities was not linear, but depended on the bear population size, as determined by *Exp (-1*.*792 + 0*.0008 * bear population size). We selected four population sizes within the range of data during our study period and equal internal spacing among them, i.e., 500, 1300, 2200, and 3000 bears. The predicted number of hunter injuries/fatalities was 0.25 per year with 500 bears; 0,47 when there were 1300 bears; 0.97 for 2200 bears, and 1.84 for 3000 bears. This translates to a per capita annual risk of 0.00000695, or one injury per year over 144,000 hunters, for the 264,739 hunters in 2010, when the bear population was 3000 bears, for example.

## Discussion

The increase in the brown bear population during 1977–2016 was positively related to the number of hunters injured/killed by bears. This relationship was also apparent for nonhunters, but the null model was also plausible in this case (Table 2). Most of the injured/killed people were hunters (33 of 44), which reinforces the conclusions found earlier using a smaller dataset [15]. The annual number of Swedish hunters during the study period averaged 293,197±20,216 (SD), and the annual number of outdoor recreationists in bear counties averaged 1,098.500±20,036. The observed increase in the number of injuries/fatalities among hunters with the increase of the bear population, despite the decreasing number of hunters, supports the conclusion that risk per capita increased for hunters during our study period. The 33 hunters injured/killed by bears during our 40-year study period, divided by the average annual number of hunters, yields an annual per capita risk of 0.000003 for being injured/killed by a bear. We have no data on bluff charges or close encounters that did not end in an injury or fatality, nor data on how many bears charged, were shot at, and did not injure the hunter. Nevertheless, it is reasonable to pose that risk, as shown by above figures, is extremely low. For nonhunters, the 11 cases equal an annual per capita risk of 0.00000025, suggesting that hunter’s per capita risk is about 12 times higher than that of nonhunters.

Swenson *et al*. (1999) [15] concluded that the most dangerous situations regarding bear attacks involved a wounded bear; the presence of a dog was also common. In the expanded 1977–2016 data, injured bear hunters had shot at the bear before being injured in most incidents (83%) and bear-hunting dogs were present in all but one incident (92%). In comparison, injured hunters hunting for other game species shot at the bear less often (in 63% of the incidents) and hunting dogs were present less often in these incidents (in 63% of the incidents). Although this may suggest that dogs exacerbated the situation, we did not have enough data to determine whether this was the case or how it may have occurred.

The number of incidents involving bear hunters was comparatively low (12 incidents among the 33 injured/killed hunters), which means that one should use caution when interpreting the numbers for this particular group. Lack of a specific bear hunting license makes it difficult to estimate how many hunters specifically targeted bears. However, interest in bear hunting has increased, perhaps as a response to the higher bear harvest quotas. The proportion of bears killed by bear-oriented hunters increased from 46% in 1981–2004 to 71% in 2005–2012, and more hunters had specialized in bear hunting in the latter period [24]. It is therefore not surprising that bear hunting is a riskier activity than other hunting or nonhunting activities, because the probability to encounter a bear at close range should be higher for bear hunters. Nevertheless, given that many bears are shot by people hunting primarily moose [28], we used the annual number of Swedish hunters as the only available proxy for the total numbers of hunters during our study period.

Because of their high vulnerability, educating hunters (see S1 File) should help reduce the number of bear-caused human injuries/fatalities in Scandinavia. Educational campaigns to that effect started in 2007 and there was a reduction in attacks after 2008, despite a high level of bear harvest (Fig 1). A longer series of time with ongoing education campaigns may be necessary to statistically document whether education reduces the number of incidents. Using education to reduce injuries/fatalities caused by large carnivores also has been proposed elsewhere [6,35], but the effects have rarely been documented [36].

It seems important that educational and information campaigns be aimed at hunters, because the clearest result of our study is that hunters’ risk of injury increased with an increasing bear population size. Although hunters are specifically vulnerable to bear attacks, they are also an easily identifiable group. We suggest that education campaigns be intensified, especially for hunters, but also for other outdoor recreationists, where large carnivore populations are recovering in former ranges in human-dominated landscapes [10,11]. Hunters and outdoor recreationists are present in landscapes that now have bears and other carnivores, but did not have them quite recently.

Half of the hunters injured/killed by bears were initially hunting moose, often sneaking toward a barking dog thought to be holding a moose at bay in dense vegetation, a common moose-hunting technique in Scandinavia [28]. Combining moose and bear hunting using dogs that pursue both prey species is also common in Sweden. The majority of the incidents happened between 08:00 and 16:00, when bears are found primarily in dense vegetation [37]. This would explain why the hunters were first aware of the bear at short distances and also why so many of them shot at them at short distances. All of these factors increased the risk of injury for hunters and probably explained why hunters dominated among the incidents (33 of 44), even if the annual number of hunters was much lower than the number of nonhunting outdoor users.

More than half the casualties involving hunters occurred after 1 October (Fig 3), even though hunting activity is less than in late August and September. This coincided with the end of hyperphagia, and the bears’prehibernation and denning periods [38]. At that time, bears may be more likely to respond aggressively to disturbance, because of their more lethargic prehibernation behavior [39]. The bears’ activity level, heart rate, and body temperature drop slowly several weeks before denning, and bears enter the den when snow arrived and ambient temperature reached 0°C [40]. In another study, half of the bears significantly reduced their activity before arriving at the den site: on average 2,169 m from the den and 1.8 days before arrival, whereas the other half reduced their activity after arriving at the den site [39]. Denning starts at the time of the year when many moose hunters accompanied by dogs are in the forest and the bear quotas have been filled. Dog breeds traditionally used for moose hunting in Scandinavia readily pursue bears in addition to moose. Bears may react more aggressively to disturbance at that time, not because they are defending themselves at or near a den, but because their reduced activity may prevent them from using escape as a defensive mechanism [40], which they usually do otherwise [41]. Most of the incidents involving hunters probably could have been avoided if the moose hunters had been more aware of the possibility of the presence of a bear when sneaking towards a baying dog in dense vegetation. This is especially relevant after about 1 October, when bears start denning [38]. Therefore, it is important that hunters and other outdoor recreationists understand the factors driving bear denning, so everybody knows that meeting a bear can be more risky after the beginning of October.

Bear attacks on nonhunters are much rarer (n = 11 in 1977–2016) than for hunters and trends can therefore be harder to detect. We found an increasing trend in attacks for nonhunters in relation to the increase in bear population size, but the null model was also among top-ranked models (Table 2). In any case, the risk of a bear attack is particularly tiny for nonhunters. The Scandinavian brown bear is considered to be less aggressive than its North American counterpart [42]. The 2 fatalities in 39 years in Scandinavia (1977–2016) contrasts, for instance, with the 55 fatalities caused by brown bears in Alaska in 135 years (1880–2015) [43], i.e., there was one fatality every 19.5 years in Scandinavia vs. one fatality every 2.5 years in Alaska. However, the brown bear population is ~10-times larger in Alaska [43] than in Scandinavia, where human population density also is higher. Recent research on bears in Scandinavia shows indeed that bears avoid humans, both spatially and temporally, by living in rugged terrain far from human settlements [44–46], being active at night [47], especially when hunting starts [48,49], and resting in dense vegetation cover during the day [37]. More than 500 experimental encounters between bears and hikers at a distance of approximately 50 m have been conducted in Sweden [41,50]. None of the bears involved reacted aggressively toward the researchers; rather they ran away and/or hid in dense vegetation [41,50]. Although food-conditioned or habituated bears is a common factor in incidents between bears and people in North America, particularly in national parks [51], our results suggest that the nature of bear attacks in Scandinavia is defensive. Furthermore, the body condition of bears shot in self-defense in Sweden (no injuries/fatalities involved) was not different from that of hunter-killed, nonproblem bears [52], suggesting that reasons other than food shortage or malnutrition was involved in these incidents.

Very few people experience an actual encounter with brown bears in Scandinavia and the risk of people involved in outdoor nonhunting activities being injured by bears is exceedingly low. Nine of 10 nonhunters injured with known time occurred during the daylight hours and the bears were encountered at much shorter distances (11±9 m) than the normal flight initiation distance of resting bears (69±47 m; [41]), suggesting that the bears might have been surprised when in daybeds or unable to flee. Eight of the 11 incidents (73%) involving nonhunters were caused by female bears with cubs, which they probably defended when surprised; three of these family groups were in dens and two were on carcasses.

Understanding the factors involved in carnivore attacks on people allows the formation of recommendations for how people should behave during encounters with bears to reduce the risk of a negative outcome, and also inform people about what to expect from the bears. These are two important aspects of reducing fear [53,54]. Mitigating risk factors is essential for ensuring the coexistence of people and the increasing large carnivore populations in human-dominated landscapes. Although bear-caused injuries/fatalities continue to be very rare events, they also have occurred recently elsewhere in Europe, e.g., in Finland (first fatality since at least 1936) [55], Greece (first fatality ever documented there) [56], Turkey (3 lethal bear attacks and several nonlethal incidents documented since the early 1970’s) [57], and Russia, where there seems to be an increasing number of bear attacks in recent times [13]. It is thus essential that managers and the public have accurate and correct information regarding the risks that carnivores pose to people and how these risks can be reduced. Ensuring human safety is essential for the acceptance [58] and survival [59] of large carnivores, which are often persecuted because of the real or perceived threat they pose to people, both in terrestrial [3] and marine ecosystems [60].

## Conclusions

Close monitoring of the trends in bear-caused casualties and a continued focus on education campaigns using knowledge of previous incidents especially aimed at hunters, but without forgetting the general public and people involved in forestry activities, can be important to reduce the number of future bear-caused injuries/fatalities in Scandinavia. This is key for both human safety and large carnivore conservation, and should therefore be a major management task in the human-dominated landscapes where most large carnivore populations live nowadays.

We recommend that hunters be educated to be prepared for the risk of encountering bears when stalking other prey when using dog breeds that may pursue or bay at bears, especially in late fall, when bears may be more prone to attack, due to denning and lowered physiology. Bear hunters or hunters combining moose and bear hunting should be aware that shooting at charging bears or at bears that are agitated due to the presence of a barking dog at very short distances probably is risky. We could not estimate the level of this risk, however, because most of the bears that hunters shot at had already begun their charge before the hunter shot at them, and we do not know what would had happened had they not fired the shot. Only highly skilled personnel should pursue wounded bears. In North America, bear spray is a very efficient deterrent of bear attacks [61], and its potential use in areas with growing populations of bears in Europe should be studied and perhaps implemented. However, it is presently illegal to use in Scandinavia. As safety measures for both people and bears, outdoor recreationists (e.g., hikers, berry pickers) and forestry workers should avoid dense vegetation in rugged terrain or make noise (e.g., talk loudly) when entering patches of forest with low visibility to warn any bears of their presence [37,50,62].

## Supporting information

S1 File Summary from the course “Säkrare björnjakt”. (DOCX)
