# Supplementary material for: Brown bear (Ursus arctos) attacks resulting in human casualties in Scandinavia 1977–2016; management implications and recommendations
Source: PLoS One. 2018 May 23;13(5):e0196876. doi: 10.1371/journal.pone.0196876 (PMC5965840; doi:10.1371/journal.pone.0196876)
Supplement: S1 File — (DOCX) [file pone.0196876.s001.docx]

**S1 Summary from the course “Säkrare björnjakt”.**

*Swedish Hunting Association, 2008. https://jagareforbundet.se/*

The “safer bear hunting” course aimed at improving hunters’ knowledge about brown bears in the context of a growing bear population in Scandinavia. It is meant for bear hunters, but also for other hunters in the bear range. The course informs that in a normal situation, a bear detecting a person will just hide and most encounters will go undetected to the person, who will rarely see a bear. The course highlights that hunting with dogs has been involved in most incidents, as we show in the manuscript. If the dog is barking at an animal that cannot be seen by the hunter, the hunter should take into account that it might be a bear and not a moose or other targeted animal. The hunter is recommended to pay attention to non-normal behavior of the dog. The courses suggests stopping and waiting, paying attention to details, such as presence of ravens or the smell of rotten meat, which might indicated the presence of a carcass and a bear at it. The course makes specific recommendation on shooting: it should be a clean side shot, with maximum shooting distances of 80 m and using a support for the weapon. Repeat a new cartridge immediately and shoot more shots as long as the bear is on its feet. If the bear is closer than 40 m, do not shoot if you are not absolute sure to place the shot correctly. A weapon in the hand is no security in a direct confrontation with an attacking bear. The hunter must hit it in the brain, which is not much bigger than a tennis ball, which is bouncing straight towards the hunter at 45 km/h. The bear’s chest is protected with strong bones and should not be targeted. If you still have to shoot from the front, hold high and aim at the ears and the neck. Never shoot such a shot as a first shot. If a bear is shot and injured, a dog specially trained to track damaged game must be in place within two hours. If the hunting team does not have a bear hunting dog, they should have contacted potential help before the hunt is set up and that assistance must be ready to help if the case arises. The hunting team leader is responsible for organizing the search. The Hunters Association's bear hunt training includes a voluntary shooting test. The hunting team should decide that the tests will be completed annually. The shooting is individual and is actually aimed at clarifying the need for a clean side shot on the first shot. The test is to shoot four shots in a quick succession without support at a bear-shaped target at 80 and 40 meters, . This is for the recommended situation, where the hunter targets the side of an standing bear, and also the threatening situation, where the hunter perceives that the bear is attacking and where speed is of utmost importance. The test is approved when all shots hit within the recommended hit area within the allowed time interval.

The course highlights four points as a summary:

• The bear should be standing still when it is shot from the side, achieving a killing lung hit on the first shot.

• Do not shoot longer distances than are sure to hit the bear.

• Always use support for the weapon.

• Refrain from shooting if it is not certain that the shot will be a good one.
